# Writing a Case Report in Pediatric Surgery: A Comprehensive Guideline

**DOI:** 10.1055/s-0041-1740935

**Published:** 2022-02-10

**Authors:** Xiaoyan Feng, Richard Wagner, Silvia Rogers, Martin Lacher, Ophelia Aubert

**Affiliations:** 1 Department of Pediatric Surgery, University Hospital Leipzig, Leipzig, Germany; 2 Department of Pharmaceutical Sciences, University of Basel, Basel, Basel-Stadt, Switzerland

**Keywords:** case report, guidelines, pediatric surgery, CARE guidelines, rare disease

## Abstract

An effective case report delivers clear and valuable clinical or surgical information to the medical community. Case reports dealing with pediatric surgical issues raise the medical community's awareness of rare diseases, unusual presentations of common disorders, or novel surgical or nonsurgical treatment approaches. Thus, case reports contribute substantially to medical advance by sharing remarkable or unexpected findings. For this reason, case reports should be prepared with vigilance, and current conventions on good medial writing practice should be observed. This guideline aims to assist clinicians and surgeons in the successful publishing of a compelling case report in pediatric surgery that is read and understood by the intended audience.

## Introduction

### What Is a Case Report?

A case report is an account of anecdotal evidence and is therefore usually located at the bottom of the hierarchy of clinical evidence. 1 However, case reports are of substantial value to the scientific community. They enable scientists and physicians to share information on unknown illnesses, unexpected outcomes, or novel treatment approaches, thereby broadening current medical knowledge. 2 In addition, case reports are often the first publication format encountered when researching an atypical clinical situation. In the field of pediatric medicine and surgery, case reports represent an important platform for sharing information on the presentation of rare disorders, atypical course of illnesses, novel surgical or nonsurgical treatment approaches, and associated complications.

To ensure effectiveness of a case report, the text should be presented as a “story” which readers will want to read. 3 This implies an enticing opening statement and finally a logical conclusion that ties in with the initial query. The part between the beginning and ending of the report must guide the reader smoothly from one statement to the next thus constituting a “red thread.”

Although case reports do not have statistical power, their publication can be educational for authors, as well as readers. As early as 1920, Sir William Osler, one of the four founders of the Johns Hopkins Hospital located in Baltimore, Maryland, United States, stated, “Always note and record the unusual …. Publish it, place it on permanent record as a short, concise note. Such communication is always of value ….” 4 Writing a case report is not only an excellent way to improve the writing skills of medical scientists new to publishing but it also provides first-hand insight into the critical peer-review process. 5

We aimed to provide a guideline on how to write an engaging and pertinent case report in pediatric surgery, both in terms of structuring the contents, as well as getting the messages across effectively. In each section, we cited example case reports retrieved from the *European Journal of Pediatric Surgery Reports* to illustrate the recommendations. Furthermore, a concise checklist provides an overview of the main recommendations ( Table 1 ).

**Table 1:** Checklist for a case report in pediatric surgery

Topic	Item		Checklist
Total manuscript	1		Number of words: 1,000–1,500
Title	2	a b c	Number of words <20 Pinpoint the topic of interest Include the phrase “case report”
Keywords	3		3–5 keywords
Abstract	4	a b c d e	Number of words: around 150 Structure: introduction, case description and conclusion No discussion Finish with one sentence outlining the “take-home” message Do not list subheadings like: “introduction,” “methods,” “results,” and “conclusion”
Introduction	5	a b c d e f	Number of words: 10% of the total manuscript (100–150) Briefly introduce the context, the controversies to attract reader curiosity Include the number of already published cases on the topic and outline the rarity of the reported case Conclude with the introduction of the reported case: e.g., “We present a case of ….” Cite 3–5 key references Do not be too detailed (it is not a review)
Case description	6	a b c d e f g h i j	Number of words: 40–45% of the total manuscript, depending on the case Present only relevant patient information For neonatal surgical cases include gestational age, birth weight, Apgar's score, perinatal status, maternal health etc. Important positive/negative findings on physical examination for differential diagnosis Diagnostic workup, relevant preoperative imaging studies: e.g., X-ray, ultrasound, computed tomography/magnetic resonance imaging, angiography, endoscopy Decision-making process leading to surgery/intervention Detailed report of the surgical technique/intervention Intraoperative findings, pictures/videos: e.g., screenshot of endoscopic procedures/surgery Postoperative outcomes/complications, histopathological findings: gross view/microscopic/specific marker Important follow-up information
Discussion	7	a b c d	Number of words: 40–45% of the total manuscript, depending on the case • “rare” case: focus on case description rather than discussion • common disease with unusual course of illness: focus on discussion rather than case description Create a flowchart or subheadings to structure the discussion, and delete them afterwards Discuss only the relevant aspects in the context of relevant literature Avoid redundancies
Conclusion	8	a b c d	1–2 sentences Provide a “teaching value“ or “take home” message Do not use words such as “could,” “might,” “probably” Avoid overgeneralization
References	9	a b	Cite no more than 20 publications Cite only the most recent and relevant publications (<5 years, exceptions: landmark publications)
Tables and figures	10	a b	Use accurate and concise legends for all tables and figures Make sure to hide all information that could identify the patient
Informed consent	11	a	Obtain written informed for consent of patient/legal guardian when using patient identifying materials
Language	12	a b	The manuscript should be written in American English and revised by a native speaker Alternatively, use a professional language editing service (oftentimes offered by the journal of submission)

Our instructions are intended to complement the established CARE (CAse REport) guidelines and checklist published in 2017. 3 Notably, our guidelines should be used in conjunction with the “Instructions for Authors” of the journal chosen for publishing the case report. Although the structure and formats of case reports have been harmonized across journals in the past years, some small differences may still exist.

### Structure of a Case Report

#### General Aspects and Sequence of Writing

On average, a case report contains 1,000 to 1,500 words and consists of the following five parts: (1) abstract, (2) introduction, (3) case description, (4) discussion, and (5) conclusion.

Before starting to write the case report, authors should make sure to have completely understood the “story” they wish to share. In terms of the sequence of writing various sections of the paper, it may be tempting to start with the abstract or introduction, but this tends to lead to insufficiently focused abstracts and overly lengthy introductions. A more effective approach is to start with the case description including tables and illustrations. We recommend the following procedure:

1. Prepare the figures, tables, photos, etc.
2. Write the case description.
3. Write the discussion.
4. Write a clear and concise conclusion.
5. Write a compelling introduction.
6. Write the abstract.

The text below outlines the main points to consider for each part of the case report.

#### Title

The title draws immediate attention to the nature of the case report. Consequently, the title must be specific but concise, containing no more than 20 words or 85 characters. The term “case report” should be included in the title. 3 6

#### Keywords

For a case report, three to five keywords are generally recommended. 7 Use key words specific to your case and anticipate the keywords other researchers are likely to select when looking for the type of information you provide in your case report. Careful selection of keywords will generate more reads and citations of your case report.

#### Abstract

A good abstract can be retrieved easily from an online database. The abstract should be kept short (usually ≤250 words, but word limit may vary depending on the target journal). Start with a brief introduction of the subject limited to approximately three sentences, followed by a short description of the case, including diagnostic workup, treatment, and outcome. Finally, provide a concise conclusion emphasizing the teaching value. 3 Point out the uniqueness of the case and in what way it contributes to the existing literature in the field of pediatric surgery. Many journals (e.g., *European Journal of Pediatric Surgery Reports* ) do not use the standard subheadings (i.e., Introduction, Methods, Results, and Discussion referred to as IMRaD) in the abstract. 8 9 However, other journals encourage IMRaD even in the abstract. Thus, consult the “Instructions for Authors” of the target journal before you write the abstract.

#### Introduction

The introduction should be concise and attract the readers' interest. It should contain 100 to 150 words and should not exceed 10% of the entire manuscript. Assume that readers are reasonably familiar with the topic, thus refraining from providing unnecessary detail that may overlap with the discussion. 10 11 Highlight the rarity of the reported case and include the number of similar cases already published. The “3C” technique can be used to structure the introduction. Describe each “C” with 1 to 2 sentences. The first “C” stands for “condition.” Here, provide general background information on the disease, diagnostic and therapeutic procedures if available, and possible assessment of outcomes. The second “C” stands for “controversy,” referring to current debate or ongoing discussion of the topic as the reason for writing the case report. The last “C” stands for “closure.” A sentence such as, “We present a case of …” can be used to conclude the introduction. 12 In terms of literature references, cite three to five key references in this part of the case report.

As pointed out above, the introduction should be written after completing the case description. If you start with the introduction, this section tends to be less focused and may be excessively long.

#### Case Description

The description of the case is the most important part of a case report. Depending on the case, this section should account for 40 to 45% of the total length of the manuscript. To facilitate its compilation, collect detailed information and imaging materials before writing this section. Write in the past tense since the reported case occurred in the past (see also Table 2 ). Highlight the unique and atypical findings in line with their sequence of appearance. This includes patient demographics, chief complaints, positive/negative findings on physical examination, diagnostic workup resulting in the primary diagnosis, and potential differential diagnoses. Moreover, describe the decision-making process leading to an intervention or operation, intraoperative findings, final diagnosis, postoperative outcome, and follow-up. 13 14 15 Give detailed information on the operation or intervention (e.g., operation time, type, and number of trocars or sutures).

**Table 2:** The “tense” rules

Part of paper/nature of information	Tense
Established knowledge, previous results, generally known facts	Report in the present tense
Methods applied, materials used in the current study	Report in the past tense
Description of results	Report strictly in the past tense
Description of tables, figures, and other displays	Refer to them in the present tense because they are part of the actual case report
Attribution	Refer to other researchers in the past tense, e.g., Rendall et al reported similar findings

Note: Adapted from Rogers SM. 26

Where appropriate, provide some information on the effects on the patient and his/her family or caregivers. In cases of neonatal surgery, address prenatal findings and interventions, provide information on gestational age, mode of delivery, birth weight, Apgar's score, perinatal status, and maternal health if relevant. 16

The saying, “A picture says more than a thousand words” holds true, especially for surgical cases. Therefore, we strongly recommend the use of preoperative imaging studies, intraoperative pictures/videos, or images of histopathologic examinations. 8 17 18 19 With smartphones readily available, photo and video documentation has been further facilitated. 20 21 Obtain written permission from the patient (if appropriate), parents, or caregiver to use medical information and images. Make sure that all patient identifying information has been deleted from images.

#### Discussion

The discussion is a key part of the case report, usually constituting 40 to 45% of the total length of the manuscript. Here, you should state why the reported case is unique. The discussion should integrate the findings in the context of current literature related to the reported case. 22 23 If the case concerns a rare disease, focus primarily on the case description. If the case deals with a known disorder with atypical presentation, course of illness, and/or complication place main emphasis on discussing these phenomena. However, limit the level of detail and avoid redundancies. Remember that a case report is not a review article.

Finally, summarize the study strengths and limitations 3 and thoroughly discuss any unexpected outcomes including complications. 24 Emphasize any new medical knowledge gained and the reasons for making this case worthy of publication.

When drafting the discussion, you may find it helpful to use a flow chart or specific subheadings to structure the section. Delete these subheadings in the final version of the manuscript. As pointed out above, the discussion should be written *after* completing the case description but before writing the introduction. This helps to limit unnecessary overlap with the introduction.

#### Conclusion

The conclusion should be brief and evidence based. Point out the teaching value of the case in one or two clear statements. 18 Avoid overgeneralization and the use of vague terms such as “probably,” “potentially,” “might,” or “seem.” 25

#### References

Limit the number of references to 20. Cite only the most recent (<5 years, exceptions can be made for landmark publications) and pertinent publications.

#### Tables and Figures

Tables and figures add useful information, but their numbers should be limited. Before compiling the displays, consult the “Instructions for Authors” of the target journal with respect to the numbers allowed in a case report. Refer to all displays in the appropriate part of the text. Add comprehensive and concise captions to each display to render each of them self-explanatory. Delete patient identifying information from all displays.

## Formal Aspects of a Case Report

### Grammar Issues

A poorly written case report will fail to convey the pertinent message because it may not be read or understood by the intended audience. Thus, the quality of writing plays a critical role in preparing an effective case report. For many scientists and clinicians, English is not their native language which adds to the challenges of clear and concise writing. When drafting a case report, nonnative, as well as native, English speakers benefit from focusing on the most important rules governing issues of grammar and style. 26 27 Table 3 lists the most common trouble makers encountered when preparing a case report or other type of publication.

**Table 3:** The most common issues in science writing

Issue	Comments
Choice of tense	The choice of tense is critical to the meaning in English science writing, contrary to other languages (e.g., German). The present tense indicates known facts or established knowledge or both. The past tense indicates new findings, including your own.
Passive writing	Language uncertainties may predispose to passive writing. Passive writing tends to make statements longer and vague; thus, active writing almost always conveys the message more readily.
Positioning adjectives and adverbs (syntax)	Adjectives used in a series usually follow a specific order: (1) article, (2) judgement, (3) size, (4) age, (5) shape, (6) color, (7) nationality, and (8) material. Thus, “a tall, 20-year-old French patient” would be correct. Keep adverbs close to the verb to avoid confusion. In the past, placing an adverb between the infinitive (known as a split infinitive; e.g., to quickly determine) was not acceptable practice. Nowadays, you may split an infinitive if you have a good reason for placing the main emphasis on the nature of the action (e.g., to randomly allocate patients to treatment groups). In most situations, however, we are still on safer ground when placing the adverb after the verb.
Dangling modifiers	Dangling modifiers do not correctly modify the subject of the sentence. A special case of a dangling modifier is the dangling participle. Consider the following: Determining the minimal inhibitory concentrations (MICs) of the antibiotics, the novel compound proved more effective than the comparators. The participle “determining” at the beginning of the sentence implies that the novel compound determined the MICs of the antibiotics, while, in fact, we determined them. Dangling participles often form the first word in the sentence. If this “ing” word does not tie up with the subject of the sentence (here “the novel compound”), we create a dangler that is very likely to distort (if not destroy) the intended message.
Wordiness	The tendency to use two or three words when one would do is common. Quantity does, however, never compensate for quality, and short sentences help to make messages clear.
Definite versus indefinite article	The only definite article in the English language (the) is used when referring to something known by both the writer and reader. If you discuss a specific method, you would refer to it as the method. In contrast, the indefinite articles (a or an) are used with nouns that are not specific. For example, you may develop a new method that subsequently becomes the method in your text. A is used for words that begin with a consonant sound (e.g., a method), and an is used for words that begin with a vowel or vowel sound (e.g., an analysis or an hour).

#### Simple Tenses Applied Properly

The main tenses used in science reporting are the present tense and past tense, with other tenses, such as the perfect tenses or future tenses, used rather sparingly. In scientific documents, the present tense indicates known facts, general knowledge, or established findings. In contrast, we use the past tense for all actions done in the context of the case report, as well as for the findings obtained. By using the past tense, you indicate to the reader that these are new, previously unpublished findings ( Tables 2 and 3 ).

#### Active Sentences Where Possible

Nowadays, most journal publishers and regulatory bodies, such as health authorities and governmental agencies, favor the use of active voice in documents disseminated to the medical community or general public. 28 The main arguments in favor of active writing is that readers have a right to know who was responsible for the work. In addition, active sentences tend to be shorter. Thus, make sure that most sentences (approximately 70%) of your case report are in the active voice. Use the personal pronoun “we” if you refer to your activities ( Tables 3 and 5 ).

**Table 4:** Comma rules

Rule	Example
Serial comma (termed Oxford comma in British English)	Scientists should exercise great care when drafting, structuring, and proof reading a manuscript.
Essential and nonessential clauses	The patients who were eligible were randomly allocated to one of the two study groups (essential clause). The patients, who had been fully informed about the study procedure, were randomly allocated to one of the two study groups (nonessential clause).
Compound sentences	We used the Wilcoxon's rank test, and all data were tabulated.
Prepositional or adverbial introductory phrases	After mixing the solvents, we added the test compound (prepositions introductory phrase). However, we only used the viable cells (adverbial introductory phrase).

Note: Adapted from Rogers SM. 26

**Table 5:** Strategies for eliminating wordiness

Tip	Examples
Avoid “there is,” “there was,” or “this is,” etc., at the beginning of the sentence. Use action verbs rather than forms of the verb “to be”	It was our intention to study the in vitro mechanism of gastrointestinal absorption (wordy). We intended to study the in vitro mechanism of gastrointestinal absorption (revised).
Use active rather than passive voice because active sentences are clearer and sometimes shorter	The results were analyzed using several statistical tests (unclear because passive). We analyzed the results using several statistical tests (revised).
Make the real subject the actual subject of the sentence; make the real verb the actual verb	In their review, there is ample evidence of the discrepancy in findings reported by the various institutions (wordy). Their review clearly documents the discrepancy in findings reported by the various institutions (revised).
Limit multiple adjectives and adverbs	The stain we observed was large, red in color, irregularly shaped, and very extended (wordy). We observed a large red stain of irregular shape (revised). The effect was very highly statistically significantly more pronounced in the second experiment than the first (wordy). The effect was significantly greater in the second than the first experiment ( *p* < 0.001) (revised; actual *p* -value given).
Avoid redundancies, e.g., in my personal opinion, for the purpose of, in an attempt to, at the present time, etc.	At the present time, there are no guidelines with regard to quality assurance of these proteins as far as we know (wordy). Currently, no known guidelines exist for the quality assurance of these proteins (revised).
Delete unnecessary phrases and clauses, e.g., in the event that, due to the fact that, the reason why is that, etc.	Because of the fact that there were many leaking cells in the event when they were incubated overnight, the incubation times were adjusted by means of shortening them for subsequent experiments (wordy). Because many cells leaked in overnight incubations, we shortened the incubation times in subsequent experiments (revised).

Sometimes, the excessive use of the personal pronoun “we” renders the text stilted. In such situations, mixing active sentences containing “we” with those not containing “we” tends to be the best approach. An example could be this: “We examined … (active).” “The results showed … (active).”

#### Proper Punctuation

When drafting a case report, proper use of punctuation marks greatly facilitates the writing and structuring of the text. In addition, readers will find it considerably easier to follow your train of thought since punctuation marks are devised to eliminate ambiguities and confusion. Table 4 shows the rules governing the use of commas, these being the most abundant punctuation marks in scientific and medical texts. Authors should also familiarize themselves with the proper use of other punctuation marks, especially hyphen. 26 27

### Style Issues

#### Clear Concept of the Message (“Story”)

As pointed out above, your case report should tell a “story” that peers will want to read. This requires an enticing opening statement in the introduction, followed by a logical train of thought (“red thread”) that eventually leads to a plausible conclusion.

Wordiness and poor word order (syntax) get in the way of clear presentation of the message ( Tables 3 and 5 ). A standard sentence, as defined by a rule of thumb, should be no longer than 15 words but this is not always easy to achieve in complex medical and scientific texts. However, consistent use of the strategies to reduce wordiness will help to limit sentence length ( Table 5 ).

In terms of word order (syntax), remember to keep modifiers as close as possible to the term they modify ( Table 3 ). A particularly damaging form of poor word order is referred to as “dangling modifiers.” Among these, “dangling participles” are best known and most feared ( Table 3 ). Dangling participles do not correctly modify the subject of the sentence, thus seriously distorting the intended message.

#### Consistent Spelling, Numbers, and Capital Letters

Is it “tumour” or “tumor?” Is a substance “labelled” or “labeled?” The answer depends on whether you use British or American English. While most journals permit both forms of spelling, American English has increasingly become the language of choice. Whatever spelling mode you choose, it is of critical importance to be consistent within the case report or other manuscript intended for publication. This requires that authors know the spelling differences and format the text in accordance with the chosen language.

In terms of numbers, the trend is to use numerals rather than words. Thus, many publishers opt for numerals even for small numbers, that is, those below 10 could, in principle, be spelled out as a word. Remember, however, that numerals at the beginning of a sentence are strictly disallowed; here, you spell the number as a word or rearrange word order.

In case reports and other forms of publication written by authors whose native tongue is not English, capital letters tend to be overused. In English, only proper nouns, that is, names, are capitalized whereas common nouns are not. If in doubt, you are on safer ground not to capitalize the word (e.g., generic drug name or general surgical tool) since proper nouns are rare.

In short, the magic word is consistency since inconsistent use of English, numbers, or capital letters creates an impression of careless writing which the reader may unfairly confuse with careless research.

### Final Thoughts

A compelling case report in pediatric surgery, as well as other disciplines, delivers important information on new or unexpected clinical/surgical findings. For the information to reach the intended audience, present the message as a “story” that readers will want to read. This requires that authors observe the rules of good medical writing. Bear in mind that errors and inconsistencies may jeopardize the impact of your case report.

## Notes

New Insights and Importance for the Pediatric Surgeon Our paper provides specific guidance on case report writing for pediatric surgeons, while taking the established CARE guidelines into account.
